# Proteomics identification of novel proteins dysregulated in Alzheimer’s Disease with potential as blood‐based biomarkers and related to Amyloid‐Beta Plaques

**DOI:** 10.1002/alz.086675

**Published:** 2025-01-03

**Authors:** Ana Montero‐Calle, Raquel Coronel, Vivian de los Ríos, María Jesús Fernández‐Aceñero, Alberto Peláez‐García, Javier Martínez‐Useros, Marta L. Mendes, Isabel Liste, Rodrigo Barderas

**Affiliations:** ^1^ Instituto de Salud Carlos III, Madrid, Madrid Spain; ^2^ Centro de Investigaciones Biológicas, Madrid, Madrid Spain; ^3^ Hospital Universitario Clínico San Carlos, Madrid, Madrid Spain; ^4^ Hospital Universitario La Paz, Madrid, Madrid Spain; ^5^ Fundación Jiménez Díaz University, Madrid, Madrid Spain; ^6^ Luxembourg Institute of Health, Luxembourg, Luxembourg Luxembourg

## Abstract

**Background:**

Despite being the most common cause of dementia worldwide, the mechanisms underlying the progression of Alzheimer’s disease (AD) are not clear and effective treatments are still needed. Hence, further investigation regarding the pathogenesis of AD is required, which might allow for a better understanding of the disease, as well as for an early diagnosis of AD, thus improving the clinical management of AD patients. Here, to identify key proteins in AD pathogenesis, we performed two proteomics strategies, TMT (Tandem Mass Tags) 10‐plex quantitative proteomics and LFQ (Label Free Quantification).

**Method:**

For the TMT analysis, proteins were extracted from frozen left prefrontal cortex brain tissue samples from AD at Braak IV‐VI, and from VD (vascular dementia) and FTD (frontotemporal dementia) patients, and healthy individuals as controls, trypsin digested, differentially labeled with the TMT reagents, and analyzed by tandem mass spectrometry coupled to liquid chromatography (LC‐MS/MS) using a Q‐Exactive mass spectrometer. For the LFQ analysis, a pull‐down with *in vitro* synthesized Abeta (Amyloid‐beta) fibers incubated with protein extracts from AD patients and healthy individuals was performed, and interacting proteins were analyzed on a Q‐Exactive. Proteomics data were analyzed with MaxQuant and the R program to identify dysregulated proteins.

**Result:**

Among the 3281 proteins quantified by TMT, 15 and 154 proteins were found statistically significant ≥1.5‐fold upregulated and downregulated in AD patients in comparison to controls, respectively. After bioinformatics analysis, 10 candidate dysregulated proteins in AD were selected for further validation by orthogonal techniques to elucidate their role as tissue‐ or blood‐biomarkers, using tissue and plasma samples of AD patients and controls. Regarding the pull‐down, 332 proteins were identified as potential interactors of Abeta fibers, with 23 out of them selected for validation by WB and immunofluorescence.

**Conclusion:**

The dysregulation of selected targets in AD patients was confirmed at protein level in a different cohort of tissue samples. Importantly, two proteins showed potential as blood‐based diagnostic biomarkers of AD, and eighteen candidate proteins were validated as novel Abeta plaques interactors, highlighting a major role of these proteins in AD development and progression.

